# The role of stroke-induced immunosuppression as a predictor of functional outcome in the neurorehabilitation setting

**DOI:** 10.1038/s41598-024-58562-1

**Published:** 2024-04-09

**Authors:** Gloria Vaghi, Andrea Morotti, Elisa Maria Piella, Micol Avenali, Daniele Martinelli, Silvano Cristina, Marta Allena, Valentina Grillo, Michele Corrado, Federico Bighiani, Francescantonio Cammarota, Alessandro Antoniazzi, Federica Ferrari, Federico Mazzacane, Anna Cavallini, Anna Pichiecchio, Elisa Rognone, Luca Martinis, Luca Correale, Stefano Filippo Castiglia, Dante Trabassi, Mariano Serrao, Cristina Tassorelli, Roberto De Icco

**Affiliations:** 1 Department of Brain and Behavioral Sciences, University of Pavia, Via Mondino 2, 27100 Pavia, Italy; 2 Movement Analysis Research Section, IRCCS Mondino Foundation, Pavia, Italy; 3 Neurology Unit, Department of Clinical and Experimental Sciences, University of Brescia, Brescia, Italy; 4 Department of Continuity of Care and Frailty, ASST Spedali Civili, Brescia, Italy; 5 Department of Emergency Neurology and Stroke Unit, IRCCS Mondino Foundation, Pavia, Italy; 6 Neuroradiology Department, Advanced Imaging and AI Center, IRCCS Mondino Foundation, Pavia, Italy; 7 Sports Science Unit, Department of Public Health, Experimental Medicine and Forensic Sciences, University of Pavia, Pavia, Italy; 8 Department of Medical and Surgical Sciences and Biotechnologies, “Sapienza” University of Rome, Latina, Italy; 9 Movement Analysis Laboratory, Policlinico Italia, Rome, Italy

**Keywords:** Neuroscience, Neurology

## Abstract

Stroke affects the interconnection between the nervous and immune systems, leading to a down-regulation of immunity called stroke-induced immunosuppression (SII). The primary aim of this study is to investigate SII role as a predictor of functional, neurological, and motor outcomes in the neurorehabilitation setting (NRB). We conducted a prospective observational study enrolling post-acute stroke patients hospitalized for neurorehabilitation. At NRB admission (T_0_) and discharge (T_1_), we assessed presence of SII (defined by a neutrophil-to-lymphocyte ratio ≥ 5) and we evaluated functional independence (Functional Independence Measure-FIM, Barthel Index-BI), motor performances (Tinetti Score, Hauser Ambulation Index) and neurological impairment (NIHSS). We enrolled 96 patients (45.8% females, 70.6 ± 13.9 years, 88.5% ischemic stroke). At T_0_, 15.6% of patients (15/96) had SII. When compared to immunocompetent patients (IC), the SII group was characterized by worse baseline functional independence, motor performances and neurological disability. The same was confirmed at T_1_ (FIM *p* = 0.012, BI *p* = 0.007, Tinetti *p* = 0.034, NIHSS *p* = 0.001). Neurological disability demonstrated a less pronounced improvement in SII (ΔNIHSS: SII: − 2.1 ± 2.3 vs. IC: − 3.1 ± 2.5, *p* = 0.035). SII group presented a higher percentage of infectious complications during the neurorehabilitation period (SII 80% vs. IC 25.9%; *p* = 0.001). SII may represent a negative prognostic factor in the neurorehabilitation setting. SII patients were characterized by poorer functional, motor, neurological performances and higher risk of infectious complications. ClinicaTrial registration: NCT05889169.

## Introduction

A cross-talk between the nervous and immune system is responsible for the maintenance of neuro-immune homeostasis^1^. This interplay usually counterbalances an ongoing state of peripheral inflammation with a central anti-inflammatory response^1^. The central nervous system (CNS) senses the immune state through peripheral and central mechanisms. It detects cytokines concentration changes at the peripheral level, through receptors localized on afferent vagal nerve fibers, and centrally through the passive or active flow of inflammatory cytokines across the blood brain barrier (BBB). Several relays in the CNS, mainly located in the brainstem, pituitary gland, hypothalamus and prefrontal motor cortex, process these signaling inputs and send anti-inflammatory outputs via the activation of the vagal nerve, hypothalamus–pituitary–adrenal axis and the sympathetic-adrenal-medullary axis^1^. As a consequence, brain injuries, including stroke, may alter systemically the immune system. In the case of stroke, there is also a local release of inflammatory mediators that are sensed by the CNS, thus activating the anti-inflammatory response, even in the absence of systemic inflammation. This condition is known as CNS injury-induced immune deficiency syndrome, or stroke-induce immunosuppression (SII)^1^. These events result in reduced circulating levels of lymphocytes, Natural Killer cells, granulocytes and monocytes^1–3^. The degree of these changes directly reflects the extent of brain damage^4^.

A useful and reliable tool to quantify the degree of immune response is represented by the neutrophil-to-lymphocyte ratio (NLR), which reflects the interplay between the innate and adaptive response^5^. NLR normally ranges from 1 to 2^5^. Previous studies found higher NLR values in the acute phase of ischemic or haemorrhagic stroke^6–9^. The clinical role of NLR in patients with acute stroke admitted to the intensive care setting has been detailed in several recent systematic reviews and meta-analyses^10–14^. Although a definite consensus is lacking, the presence of SII seems consistent in patients with a NLR ≥ 5^9,15–17^. This cut-off was supported by recent studies demonstrating higher mortality and infectious complications in stroke patients with a ≥ 5 NLR^9,15–17^. Altogether, this data consistently suggests that NLR ≥ 5 may be considered a proper marker of SII, associated with post-stroke mortality, short-term global outcome (modified Rankin Scale), intracerebral haemorrhage (ICH), rate of infectious complications, and post-stroke depression^10–14^. The majority of available evidence on SII is derived from acute phase studies. Conversely, the clinical role of SII in the neurorehabilitation setting, and its association to functional independence and performances in motor and daily activities, has yet to be elucidated.

The aim of this study is to investigate the potential role of SII in the functional outcomes of patients with ischemic or haemorrhagic stroke undergoing a neurorehabilitation program.

## Results

### Study population

The final study population included 96 patients (45.8% females, age 70.6 ± 13.9 years). The stroke type was ischemic in 88.5% of patients and haemorrhagic in 11.5%. All patients presented with a moderate-severe hemiparesis. Predominant associated neurological symptoms were aphasia (31.3%), mild cognitive impairment (27.1%) and dysphagia (35.4%). Stroke volume was similar for both aetiologies: 18.9 ± 40.4 ml for ischemic events and 16.2 ± 18.2 ml for haemorrhagic lesions. Patients were admitted to the Neurorehabilitation Unit (NRB) after a mean of 7.3 ± 4.5 days from the stroke onset (index event). Up to 90% of patients were admitted to NRB within 10 days from stroke onset. Table 1 summarizes clinical and demographic features of the study population, while Table 2 details clinical and radiological features according to stroke subtype at hospital admission.

**Table 1:** Clinical and demographic features of study population at NRB admission.

	Total	IC group	SII group	*p*-value
Number of patients	96 (100%)	81 (84.4%)	15 (15.6%)	-
Clinical and demographic features				
Age (years)	70.6 ± 13.9	70.0 ± 14.2	73.3 ± 12.8	0.363
Female	44 (45.8%)	37 (45.7%)	7 (46.7%)	1.000
Days from stroke onset and NRB admission	7.3 ± 4.5	7.2 ± 4.5	8.0 ± 4.3	0.417
Ischemic stroke	85 (88.5%)	73 (90.1%)	12 (80%)	0.370
Aphasia	30 (31.3%)	23 (28.4%)	7 (46.7%)	0.224
Cognitive impairment	26 (27.1%)	24 (29.6%)	2 (13.3%)	0.342
Dysphagia	34 (35.4%)	24 (29.6%)	10 (66.7%)	**0.007**
Urinary disorders	60 (62.5%)	50 (61.7%)	10 (66.7%)	0.780
Medical history				
Atrial fibrilation	24 (25%)	19 (23.5%)	5 (33.3%)	0.517
Hypertension	86 (89.6%)	72 (88.9%)	14 (93.3%)	1.000
Diabetes	29 (30.2%)	25 (30.9%)	4 (26.7%)	1.000
Hypercholesterolemia	57 (59.4%)	48 (59.3%)	9 (60.0%)	1.000
Myocardial infarction	18 (18.8%)	15 (18.5%)	3 (20.0%)	1.000
Alcohol abuse	28 (31.5%)	25 (33.8%)	3 (20.0%)	0.372
Smoking				
Active	50 (55.6%)	39 (52.0%)	11 (73.3%)	0.274
Former	26 (28.9%)	24 (32.0%)	2 (13.3%)	
Never	14 (15.6%)	12 (16.0%)	2 (13.3%)	
Clinical scales				
Pre-mRS ≥ 3	6 (6.2%)	5 (6.1%)	1 (6.7%)	0.751
FIM	72.2 ± 26.3	75.5 ± 24.7	54.2 ± 28.0	**0.007**
Barthel index	41 ± 26	44.6 ± 25.1	21.7 ± 22.3	**0.001**
Tinetti	12 ± 10	13.2 ± 9.9	5.3 ± 8.2	**0.004**
NIHSS at NRB admission	7.6 ± 4.8	7.0 ± 4.1	11.4 ± 6.2	**0.009**
NIHSS at SU admission	8.4 ± 4.7	7.7 ± 4.1	12.2 ± 6.0	**0.005**
Relevant laboratory values				
Leucocytes, 10^9^/L	8.5 ± 2.6	8.0 ± 2.3	10.7 ± 3.1	**0.001**
Neutrophils, 10^9^/L	5.6 ± 2.3	5.1 ± 1.9	8.3 ± 2.5	**0.001**
Lymphocytes, 10^9^/L	1.8 ± 0.6	1.9 ± 0.6	1.3 ± 0.4	**0.001**
NLR	3.4 ± 1.9	2.7 ± 0.9	6.9 ± 1.9	**0.001**

*SII* stroke-induced immunosuppression, *IC* Immunocompetent patients. SII is defined as a neutrophil to lymphocyte ratio (NLR) ≥ 5. *NRB* Neurorehabilitation Unit. *FIM* Functional Independence Measure, *NIHSS* National Institutes of Health Stroke Scale, *SU* Stroke Unit.

Significance values are bold.

**Table 2:** Treatment and radiological features of vascular lesion at hospital admission.

	Total	IC group	SII group	*p*-value
**Ischemic stroke**				
Number	85	73	12	–
Number of lesions				
Single	60 (70.6%)	51 (69.86%)	9 (75%)	1.000
Multiple	25 (29.4%)	22 (30.1%)	3 (25%)	
Side of lesion				
Left	46 (54.1%)	39 (53.4%)	7 (58.3%)	1.000
Right	39 (45.9%)	34 (46.6%)	5 (41.7%)	
Location				
Supratentorial	67 (78.8%)	57 (78%)	10 (83.3%)	1.000
Subtentorial	18 (21.2%)	16 (21.9%)	2 (16.7%)	
Haemorrhagic transformation	11 (12.9%)	8 (11.0%)	3 (25.3%)	0.183
Stroke volume (cm^3^)	18.9 ± 40.4	17.8 ± 39.2	25.4 ± 48.7	0.551
Treatment				
Antiplatelet	67 (78.8%)	56 (76.7%)	11 (91.7%)	0.446
Anticoagulant	39 (45.9%)	34 (46.6%)	5 (41.7%)	1.000
B-blocker	24 (28.2%)	18 (24.7%)	6 (50%)	0.089
Anti-hypertensive	33 (38.8%)	27 (37%)	6 (50%)	0.525
Statin	27 (31.8%)	23 (31.5%)	4 (33.3%)	1.000
TOAST				
Large-artery atherosclerosis	31 (36.5%)	27 (37.0%)	4 (33.3%)	0.786
Cardioembolism	25 (29.4%)	20 (27.4%)	5 (41.7%)	
Small-vessel occlusion	16 (18.8%)	15 (20.5%)	1 (8.3%)	
Stroke of other determined etiology	5 (5.9%)	4 (5.5%)	1 (8.3%)	
Stroke of undetermined etiology	8 (9.4%)	7 (9.6%)	1 (8.3%)	
**Haemorragic stroke**				
Number	11	8	3	–
Number of lesions				
Single	10 (90.9%)	7 (87.5%)	3 (100%)	1.000
Multiple	1 (9.1%)	1 (12.5%)	0 (0.0%)	
Side of lesion				
Left	6 (54.5%)	5 (62.5%)	1 (33.3%)	0.545
Right	5 (45.5%)	3 (37.5%)	2 (66.7%)	
Location				
Typical	6 (54.5%)	4 (50%)	2 (66.7%)	1.000
Atypical	5 (45.5%)	4 (50%)	1 (33.3%)	
Stroke volume (cm^3^)	16.2 ± 18.2	7.8 ± 11.7	38.4 ± 12.3	**0.024**
Treatment				
Antiplatelet	2 (18.2%)	1 (12.5%)	1 (33.3%)	0.491
Anticoagulant	3 (27.3%)	1 (12.5%)	2 (66.7%)	0.152
B-blocker	2 (18.2%)	1 (12.5%)	1 (33.3%)	0.491
Anti-hypertensive	8 (72.7%)	6 (75%)	2 (66.7%)	1.000
Statin	1 (9.1%)	1(12.5%)	0 (0.0%)	1.000

*SII* stroke-induced immunosuppression, *NRB* Neurorehabilitation department, *IC* Immunocompetent patients. SII is defined as a neutrophil to lymphocyte ratio (NLR) ≥ 5. *FIM* Functional Independence Measure, *NIHSS* National Institutes of Health Stroke Scale, *TOAST* Trial of Org 10,172 in Acute Stroke Treatment classification.

Significance values are bold.

### Prevalence of SII at NRB admission (T_0_) and factors associated with SII

At NRB admission (T_0_), 15 out of 96 patients (15.6%) had a SII. Demographic features did not differ between SII and IC groups (Table 1). Dysphagia was more prevalent in patients with SII when compared to the IC group (66.7% vs. 29.6%, *p* = 0.007). The prevalence of comorbidities and voluptuary habits was comparable between SII and IC groups (Table 1). NLR was equal to 6.9 ± 1.9 in the SII group, and equal to 2.7 ± 0.9 in the IC group (*p* = 0.001).

Subjects with SII were more disabled when compared to IC group. In particular, the SII group was characterized by lower functional independence (FIM score: SII = 54.2 ± 28.0 vs. IC = 75.5 ± 24.7, *p* = 0.007), more severe neurological disability (NIHSS score: SII = 11.4 ± 6.2 vs. IC = 7.0 ± 4.1, *p* = 0.009), lower performances in the activities of daily living (Barthel Index score: SII = 21.7 ± 22.3 vs. IC = 44.6 ± 25.1, *p* = 0.001), and lower motor performances (Tinetti score: SII = 5.3 ± 8.2 vs. IC = 13.2 ± 9.9, *p* = 0.004). According to the Hauser Ambulation Index, 52.4% in the IC group and 80% in the SII group (*p* = 0.453) needed a wheelchair for most activities (Hauser Ambulation Index ≥ 7).

NLR positively correlated with age (r = 0.204, *p* = 0.046), stroke volume (r = 0.269, *p* = 0.008), and NIHSS score at T_1_ (r = 0.315, *p* = 0.002). In addition, it negatively correlated with the scores of the following scales, both at T_0_ and T_1_: FIM (T_0,_ r = − 0.237, *p* = 0.020; T_1,_ r = − 0.209, *p* = 0.041), Barthel Index (T_0,_ r = − 0.329, *p* = 0.001; T_1_, r = − 0.206, *p* = 0.044) and Tinetti (T_0_, r = − 0.214, *p* = 0.036; T_1_, r = − 0.268, *p* = 0.008).

### SII evolution over time

**Figure 1:**
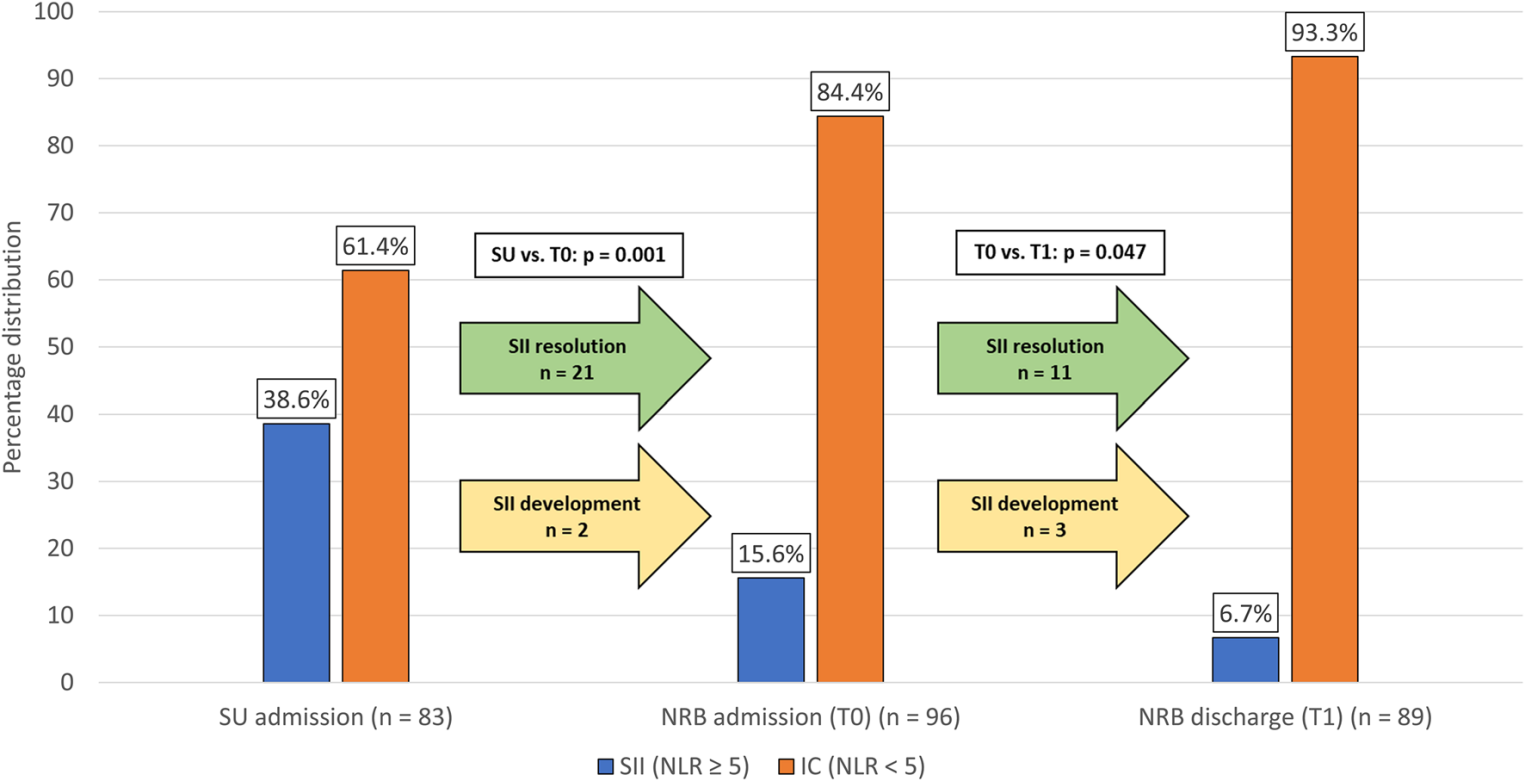
Percentage distribution of Stroke-Induced Immunosuppression (SII). Blood samples to evaluate the presence of SII were taken at SU admission (n = 83), NRB admission (n = 96) and NRB discharge (n = 89). The prevalence of SII at NRB admission was 15.6%, significantly lower when compared to the acute phase (38.6%). Only two patients received a first diagnosis of SII at NRB admission. The percentage of patients with SII at NRB discharge was low (6.7%, *p* = 0.047); noteworthy, a late-onset SII was found in three patients. SII: stroke-induced immunosuppression, defined as a neutrophil-to-lymphocyte ratio (NLR) ≥ 5. SU: Stroke Unit. NRB: Neurorehabilitation Unit.

Blood cell counts were available from 83 patients at SU admission, and from 96 and 89 patients at T_0_ and T_1_, respectively. Globally, SII showed a trend toward reduction over time. The prevalence of SII was higher at SU admission when compared to NRB admission (T_0_), (SU admission: 38.6%, T_0_: 15.6%, *p* = 0.001), and decreased upon discharge (T_1_: 6.7%, *p* = 0.047 vs. T_0_) (Fig. 1). SII resolution occurred in 21 patients between SU admission and T_0_, and in 11 patients between T_0_ and T_1_. Of note, 5 patients who were not SII at SU admission became so at later times: 2 at T_0_ and 3 at T_1_.

### SII as a predictor of the rehabilitative outcome

#### Functional independence measure – primary outcome

**Figure 2:**
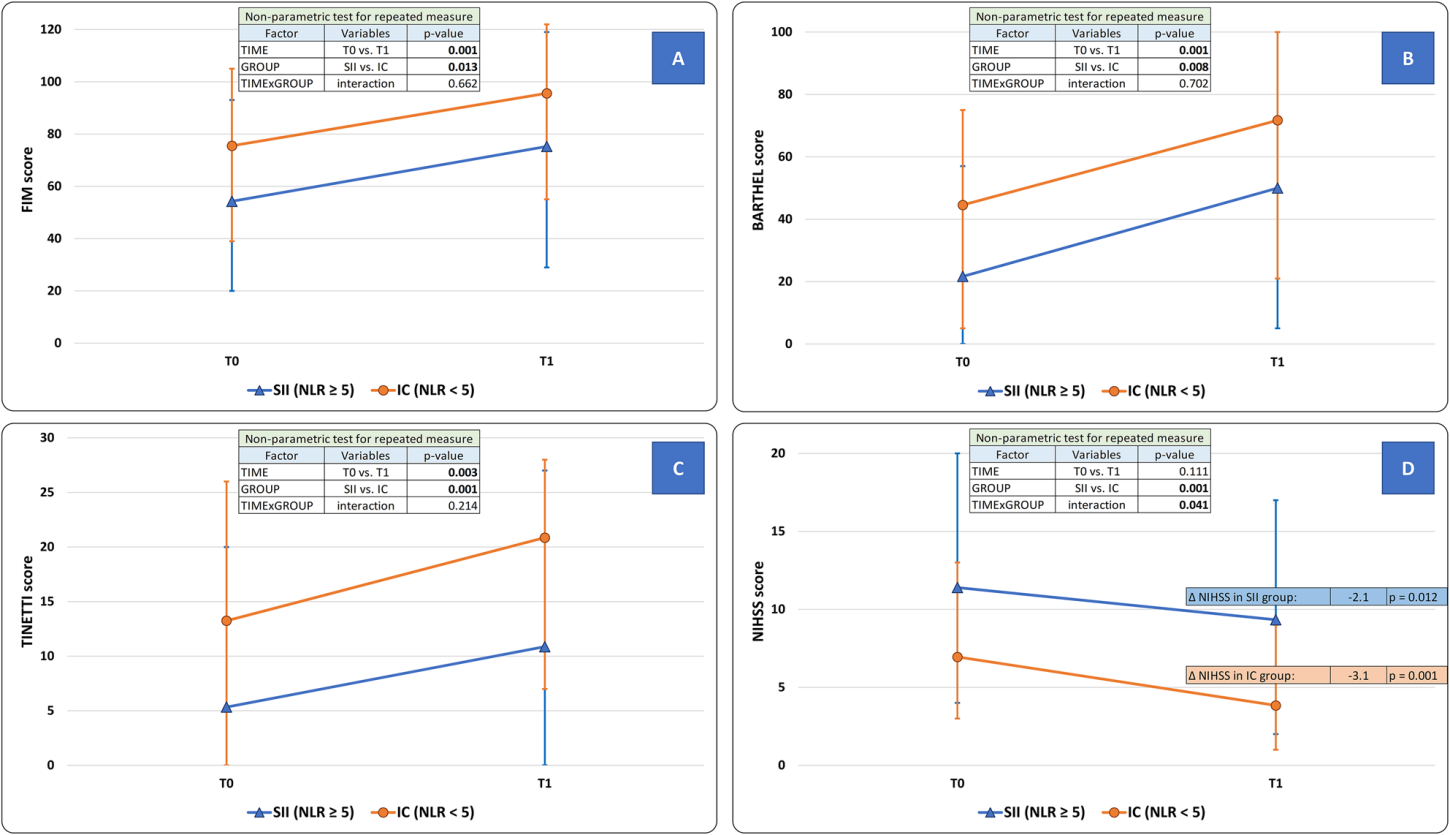
Modifications of clinical scales scores after the neurorehabilitative intervention in Stroke-Induced Immunosuppression (SII) and Immunocompetent (IC) groups. (Panel **A**) Functional Independence Measure (FIM) score; (Panel **B**) Barthel Index score; (Panel **C**) Tinetti balance scale score; (Panel **D**) National Institutes of Health Stroke Scale (NIHSS) score. FIM score (primary outcome), Barthel Index, and Tinetti scale improved at the end of the rehabilitation period (factor TIME, *p* < 0.050). For these parameters, the presence of SII was an independent predictor of overall disability (factor GROUP, *p* < 0.050), although it did not affect the degree of improvement (interaction TIMExGROUP, *p* > 0.050). Only for NIHSS score, SII qualifies as a negative predictor of the rehabilitative intervention (interaction TIMExGROUP, *p* < 0.035) as well as of the overall outcome (factor GROUP, *p* = 0.001). Statistical analysis: non-parametric test for repeated measures: factor “TIME” is expression of the efficacy of the rehabilitative treatment in the overall population; factor “GROUP” is expression of the comparison between SII and IC groups across all time-points; a significant TIMExGROUP interaction is expression of a difference between SII and IC groups as well as of a difference in the degree of improvement between SII and IC groups over time. The level of significance was corrected for “Age”, “Sex”, “Time from stroke to NRB admission”, and “Stroke volume”. SII: stroke-induced immunosuppression, defined as a neutrophil to lymphocyte ratio (NLR) ≥ 5. SU: Stroke Unit. NRB: Neurorehabilitation Unit. IC: Immunocompetent patients.

At T_1_, the functional independence (primary outcome) improved in the overall study population (FIM score: T_0_ = 72.2 ± 26.3; T_1_:92.4 ± 27.9, factor TIME: *p* = 0.001). At T_1_ however, the SII group was still more disabled than the IC group (SII = 75.3 ± 34.2 vs. IC = 95.6 ± 25.6, factor GROUP: *p* = 0.013). The extent of improvement did not differ between SII and IC groups (interaction TIMExGROUP: *p* = 0.662) (Fig. 2, panel A).

FIM score at discharge (T_1_) negatively correlated with age (r = − 0.425; *p* = 0.001), duration of hospitalization (r = − 0.448; *p* = 0.001), and vascular lesion extension (r = − 0.323; *p* = 0.001). Differences in FIM score at T_1_ were found based on the presence of aphasia at T_0_ (*p* = 0.015), presence of at least one infectious complication (*p* = 0.001) or pressure ulcers (*p* = 0.002) during hospitalization, and positive medical history of atrial fibrillation (*p* = 0.022), myocardial infarction (*p* = 0.028), or alcohol abuse (*p* = 0.023).

After statistical correction for sex, stroke volume, duration of hospitalization, days from stroke onset (index event) to NRB admission, and type of stroke (ischemic or haemorragic), the linear regression demonstrated that FIM score at T_1_ was associated with the NLR (β − 6.56 [− 11.9 to − 1.2]; *p* = 0.018), age (β − 0.58 [− 1.0 to − 0.2]; *p* = 0.010), and presence of pressure ulcers (− 26.9 [− 49.8 to − 4.1]; *p* = 0.023) (Table 3). The adjusted R^2^ was 0.568.

**Table 3:** Results of the linear regression analysis for dependent variables: Functional Independence Measure (FIM) score at T_1_.

	B parameter	95% confidence interval		*p*-value
NLR	− 6.56	− 11.9	− 1.2	**0.018**
Age (years)	− 0.58	− 1.0	− 0.2	**0.010**
Presence of pressure ulcers	− 26.95	− 49.8	− 4.1	**0.023**
Presence of at least one infectious complication	0.86	− 14.4	16.1	0.909
Sex (female)	− 5.81	− 20.7	0.1	0.429
Stroke volume (cm^3^/mL)	− 0.08	− 0.3	0.1	0.431
Stroke type (haemorragic)	− 3.13	− 16.8	10.6	0.643
Time from stroke to NRB admission (days)	− 0.41	− 2.3	1.5	0.654
Duration of hospitalization (days)	− 0.40	− 0.9	0.1	0.075
(Constant)	188.62	152.7	224.5	** < 0.001**

*NLR* neutrophil-to-lymphocyte ratio, *NRB* Neurorehabilitation Unit. In bold: parameters significantly associated with FIM score at T_1_. *T*_*1*_ Neurorehabilitation Unit discharge.

Significance values are bold.

#### Performances in the activities of daily living

At T_1_, the performances in the activities of daily living improved in the overall study population (Barthel Index score: T_0_ = 40.9 ± 25.9; T_1_ = 68.3 ± 30.2, factor TIME: *p* = 0.001). The SII group was still more disabled at T_1_ when compared to IC group (IC: 71.7 ± 27.3; SII: 50.0 ± 39.0, factor GROUP: *p* = 0.008). The extent of improvement did not differ between SII and IC groups (interaction TIMExGROUP: *p* = 0.702) (Fig. 2, panel B).

#### Motor performances

At T_1_, motor performances according to the Tinetti scale score improved in the overall study population (T_0_ = 12.0 ± 10.0; T_1_ = 19.3 ± 9.2, factor TIME: *p* = 0.003). SII group was still more disabled at T_1_ when compared to IC group (IC: 20.8 ± 7.9; SII: 10.9 ± 11.2, factor GROUP: *p* = 0.001). The rate of improvement did not differ between SII and IC groups (interaction TIMExGROUP: *p* = 0.214) (Fig. 2, panel C). Regarding Hauser Ambulation Index, SII group was still more disabled at T1 when compared to IC group. Indeed, at T_1_ 53.4% in SII group versus only 19% in IC group (*p* = 0.031) needed a wheelchair for most activities (Hauser score ≥ 7).

#### Neurological disability

Both groups achieved a significant improvement in this parameter: IC group (T_0_ = 6.9 ± 4.2 vs. T_1_ = 3.8 ± 3.1, ΔNIHSS: − 3.1 ± 2.5, *p* = 0.001); SII group (T_0_ = 11.4 ± 6.2 vs. T_1_ = 9.3 ± 5.3, ΔNIHSS: − 2.1 ± 2.3, *p* = 0.012). The rate of improvement was however more pronounced in the IC group (interaction TIMExGROUP: *p* = 0.041). The overall neurological disability was more severe in SII group (factor GROUP: *p* = 0.001) (Fig. 2, panel D).

#### Duration of hospitalization, destination at discharge and complications

The duration of hospitalization did not differ between groups (IC: 46.4 ± 15.1; SII = 51.1 ± 13.8 days, *p* = 0.081). By contrast, a lower percentage of patients in the SII group (7/15, 46.7%) were discharged at home when compared to the IC group (64/81, 79.0%) (*p* = 0.032) (Supplementary Table 1).

During SU hospitalization, 22 patients had an infectious complication. This was associated with presence of SII at SU admission as more patients with SII had a history of infectious complications during SU hospitalization (13/32, 40.6%) when compared to IC patients (9/51, 17.6%, *p* = 0.038). Conversely, infectious complications occurring in SU were not associated with the presence of SII at NRB admission. Indeed, at NRB admission only 3 patients with SII had a history of infectious complications during SU hospitalization (3/14, 21.4%) compared to 19 in the IC group (19/79, 42.1%, *p* = 0.343).

On the other hand, the percentage of patients with at least one infectious complication during NRB hospitalization was higher in the patients with SII at NRB admission (12/15, 80%) when compared to the IC group (21/81, 25.9%) (*p* = 0.001); this difference was primarily explained by the occurrence of urinary tract infections (IC: 18.5%; SII: 53.3%; *p* = 0.007) (Supplementary Table 1). The incidence of other non-infectious complications did not differ between study groups.

## Discussion

In this observational, prospective study, we aimed to assess the role of SII as a predictor of rehabilitative outcome in patients affected by the first-ever ischemic stroke or spontaneous intracerebral haemorrhage. A first observation is that SII was present in 15.6% of stroke patients who required neurorehabilitation.

Our main finding is that, while the neuro-rehabilitative intervention improved functional independence (primary outcome) in a similar extent in both groups of patients, SII patients were more compromised at NRB admission and discharge when compared with IC group. This means that the presence of SII at NRB admission did not impact functional recovery. However, the presence of SII at NRB admission had a negative impact on the final neurological disability.

The results of our study may be summarized as follows. All the evaluated disability domains were more compromised in the SII patients at NRB admission and discharge. The degree of improvement did not differ between IC and SII groups regarding functional independence, performances in activities of daily living, and motor performances.

Thus, the presence of SII at admission in the rehabilitative setting identifies a subgroup of stroke patients affected by a more severe phenotype and middle-term disability. The reduced functional independence at T_1_ observed in the SII group directly explains the lower percentage of patients discharged at home at the end of the intensive neuro-rehabilitative period.

The prevalence of SII at hospital admission was slightly lower when compared to the acute setting, where SII was diagnosed in around 25–30% of patients^8,9,18^. The differences between populations of stroke patients admitted to the acute department and the rehabilitative setting, as well as the different definition and time points of evaluations for SII, may account for the observed data. Alternatively, the difference in percentages may reflect the attenuation of the brain/systemic inflammatory response during the recovery phase of stroke^1^. This interpretation is in keeping with the further reduction in the percentage of SII patients (6.7%) at NRB discharge in our population.

In line with previous literature data, the SII group reported more infectious events during hospitalization, mainly due to urinary tract infections. Indeed, infectious diseases represent one of the most frequent post-stroke complications, affecting nearly 30% of patients during the acute/subacute phase^19^.

As reported by previous studies, we also found that stroke volume was higher in SII patients and positively correlated with NLR. Hug et al. described stroke size as a major predictor of stroke-induced lymphocytopenia 24–36 h after the index event^20^. It is worth noting that, in our population, the presence of SII, as well as NLR distribution, were predictors of the clinical outcomes independently from vascular lesion extension, as demonstrated by the multivariate analysis.

Noteworthy, a consensus on the definition of SII—and of the precise NLR cut-off for defining it—is still lacking. Possible markers of SII are represented by absolute lymphocytopenia, lymphocyte-to-monocyte ratio (LMR), reduction of monocytes HLA-DR expression, platelet-to-lymphocyte ratio (PLR)^7,8,13,18,21–23^. Park et al. showed that a low LMR was a negative predictor of good clinical outcomes (mRS 0–1 at 3 months), and it was associated to a higher predisposition to infectious complications^21^. Monocytes HLA-DR expression has been consistently reported to be associated with SII and stroke-associated pneumonia^22–25^. In past reports, NLR correlated with stroke extension and neurological severity, and it was associated with infectious complications, worse prognosis, mortality, haemorrhagic transformation and increased bleeding after treatments targeting recanalization^15,26–28^. In a recent study, when compared to 11 other parameters, NLR was superior in predicting poor prognosis in stroke patients, in particular when it was assessed on the sixth day after stroke ^29^. A NLR cut-off settled between 4.8 and 5.9 predicted mortality, infectious complications, and poor outcome at 30–90 days after stroke^15–17^. In 2019, Wang et al. evaluated 808 stroke patients from the Chengdu Stroke registry, and demonstrated that a NLR ≥ 5 was associated with haemorrhagic transformation, parenchymal hematoma, and 3-months mortality^9^. These findings corroborate the NLR cut-off adopted in our paper. A consensus definition of SII would be very useful for clinical and research purposes as it would allow a better understanding of the pathophysiology of SII, more reliable findings associated to SII between studies, and, hopefully, the identification of tailored interventions for patients with SII.

While the prospective design and the enrolment of a well-characterized stroke population, treated in an experienced intensive neurorehabilitative setting, represent important strengths of our study, we must acknowledge several limitations, which may however represent useful tips for future studies. First, our follow-up was relatively short and does not allow definite conclusions on the actual stabilized outcome of disease (6–12 months). Although powered for the primary outcome, our sample size might not have been large enough for a general transferability to the stroke population. This may be particularly relevant when considering the imbalance in IC and SII groups. It must be however noted that the prevalence of SII in the neurorehabilitation setting (15.6%) was not previously described, which prevented an evidence-based calculation. In addition, due to the lack of a globally accepted definition of SII, we cannot exclude that parameters other than NLR may represent a better marker of this condition. Lastly, we analyzed data from patients with both ischemic and haemorrhagic strokes that may have different rehabilitative outcomes and clinical evolution in the post-acute stroke phase. Nonetheless, SII condition has been described for both groups^6–9^ and our population is representative of the epidemiological distribution of stroke types. Our data could represent a starting point to design larger confirmatory studies with long-term follow-ups.

In conclusion, our findings suggest that SII is present in one out of six stroke patients admitted to the neurorehabilitation setting and may represent a negative prognostic factor for the rehabilitative outcome, at least in terms of final NIHSS score.

Identification of SII through an easy-to-use and low-cost parameter allows to identify a subset of stroke patients affected by a more severe phenotype and more at risk of developing infectious complications. Larger and confirmatory studies with a long-term follow-up are needed to clearly define the role of SII in the NRB setting.

## Methods

### Study design and subjects

This was a prospective, observational study where subjects with a recent stroke were admitted for rehabilitation at the Neurorehabilitation Unit (NRB) of the IRCCS Mondino Foundation (Pavia, Italy). The rehabilitation program lasted between 2 and 8 weeks and consisted of motor rehabilitation (500 min per week across 6 days per week) associated with speech, swallowing, cognitive and/or occupational therapy training, according to the individual clinical presentation (Supplementary material 1).

**Figure 3:**
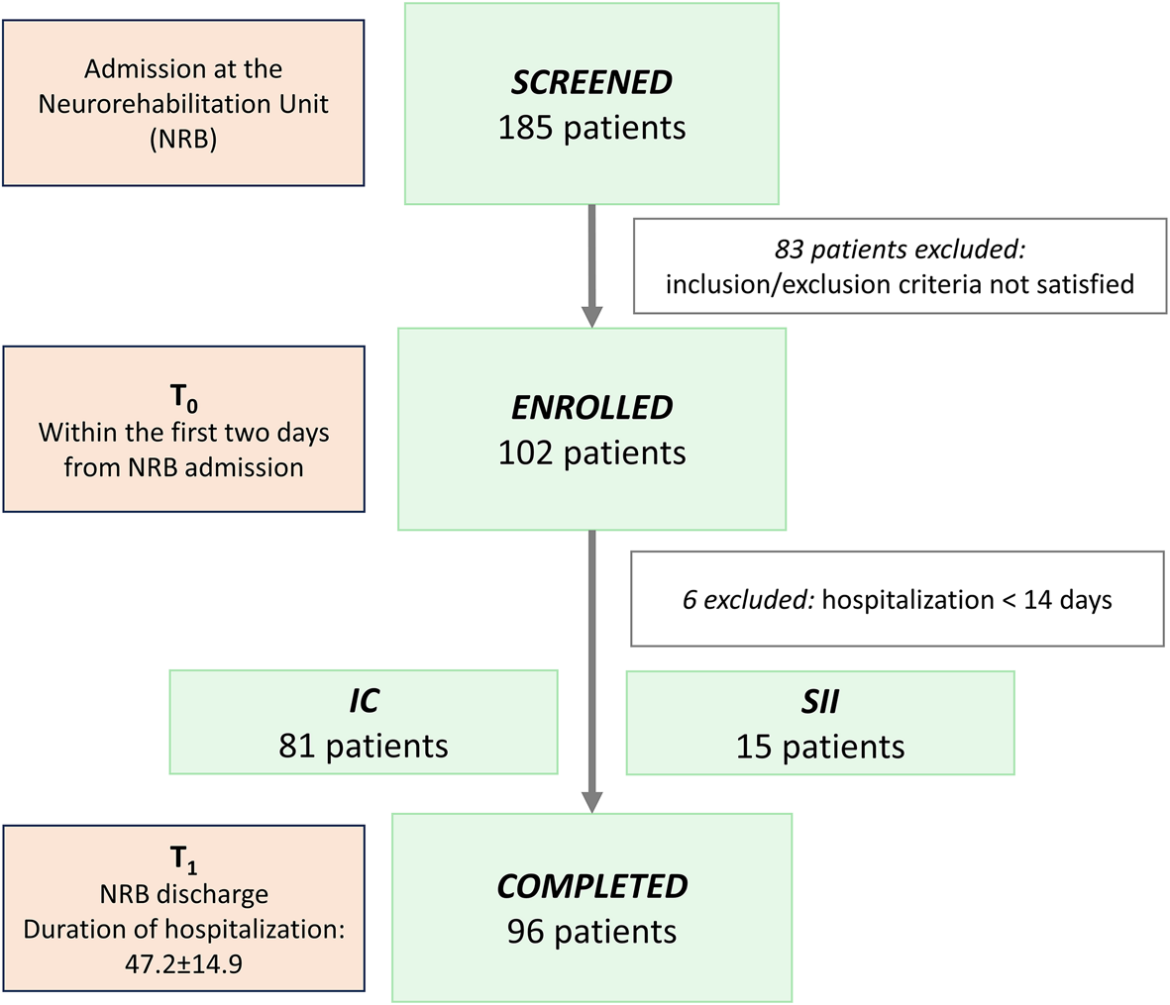
Patients’ disposition. *IC* Immunocompetent patients, *SII* stroke-induced immunosuppression, defined as a neutrophil-to-lymphocyte ratio (NLR) ≥ 5. *NRB* Neurorehabilitation Unit.

Between February 2019 and December 2021, we screened 185 subjects and a total of 96 patients were enrolled and completed the study procedures (Fig. 3).

The inclusion criteria were: i) first episode of ischemic stroke (IS) or primary spontaneous intracerebral haemorrhage (ICH) confirmed by neuroimaging; ii) admission to NRB within 30 days from the index event. Exclusion criteria were: i) age < 18 years; ii) hospitalization in the NRB < 14 days; iii) medical history of immunodeficiency or immunoproliferative disease; iv) immunosuppressive or immunomodulating therapy in the 2 years before the index event; v) intake of systemic steroids in the 6 months before the index event; vi) Glasgow Coma Scale (GCS) < 8 at hospital admission; vii) coexistence of other major neurological diseases; viii) impossibility to provide informed consent at hospital admission.

The study was approved by the local Ethics Committee (2022-3.11/58) and was registered on www.clinicaltrials.gov (NCT05889169). The procedures used in this study adhere to the tenets of the Declaration of Helsinki. The dataset generated and/or analysed during the current study is available in the Zenodo repository (10.5281/zenodo.10057752). The dataset is available from the corresponding author on reasonable request. Informed written consent was obtained by all patients before enrolment.

### Study procedures, definitions of SII and outcomes

Within the first 2 days from NRB admission (T_0_) we collected:

(i) Demographic data and pre-stroke functional status with the modified Rankin Scale (mRS);
(ii) Comorbidities;
(iii) Stroke features and associated therapy;
(iv) *Outcome scales*: Functional Independence Measure (FIM – primary outcome measure of the study), Barthel Index, National Institutes of Health Stroke Scale (NIHSS), Hauser Ambulation Index and Tinetti Balance Score.
(v) *Blood sampling* from the cubital vein for blood cell counts.

FIM is a multiple dominion scale used to assess the degree of disability in 18 items covering motor function, locomotion, mobility, personal care, sphincter control, and cognitive aspects of daily-life. Each item is assigned up to seven points according to the degree of dependence ranging from 1: completely dependent to 7: completely independent^30^.

During the hospitalization period, all patients underwent a brain imaging study (CT or MRI scan) to assess the extension of the vascular lesion. The lesion size was calculated using the ABC/2 method^8,31,32^. In addition, patients were daily monitored for ongoing complications with a specific interest in the occurrence of infectious complications. Pneumonia was diagnosed in subjects with typical symptoms of respiratory infection and confirmed by chest X-ray abnormalities^33^. Urinary tract infection was diagnosed in subjects with typical symptoms and positive urine culture without evidence of contamination^34^. Furthermore, to better detail risk factors for urinary tract infections, we also provided onset and evolution of post-stroke urinary disorders in Table 1 and Supplementary Table 1^35^. We considered as urinary disorders: urinary retention, urinary incontinence and the use of bladder catheter. Sepsis was defined as acute organ dysfunction with evidence of a clear source of infection and isolation of specific pathogens on blood cultures without evidence of contamination^36^. Other symptomatic infectious complications were diagnosed according to the clinical history with evidence of positive cultures without evidence of contamination. Patients were also constantly monitored for occurrence of pressure ulcers, localized skin or soft tissue injuries caused by long-term pressure and/or friction.

At NRB discharge (T_1_), all patients underwent a reassessment of the functional status with the same *outcome scales* recorded at T_0_, and a blood sample to evaluate NLR.

In addition, we also retrospectively collected information on blood cell counts at the time of Stroke Unit (SU) admission.

Presence of stroke-induced immunosuppression (SII) was defined as a neutrophil-to-lymphocyte ratio (NLR) ≥ 5 (SII group); accordingly, patients with a NLR < 5 were considered as immunocompetent (IC – IC group).

Our primary outcome was to evaluate whether the presence of SII at T_0_ was associated with the extent of improvement of functional independence at the end of the neurorehabilitation program (T_1_), as measured by the FIM score.

### Statistical analysis

The sample size was calculated with the online platform www.openepi.com. According to our clinical experience and based on data from the literature, we considered as clinically meaningful a difference in FIM score of 20 points^37^. For the sample size calculation, we used the following parameters: confidence interval: 95%; power: 80%; ratio of sample size (IC/SII): 4/1; between groups mean difference: 20; standard deviation: 25 for both groups. The minimum suggested sample size was 80 (64 IC patients and 16 SII patients). We planned to enroll a population of 100 patients to compensate for drop-outs and variability in expected parameters.

The statistical analysis was conducted with the SPSS software, ver. 21 (IBM Corp., USA) and with “R: A language and environment for statistical computing” (R Foundation for Statistical Computing, Vienna, Austria), Version1.2.5033, for Windows.

The Kolmogorov–Smirnov test detected a non-normal distribution of several variables, including the scores of clinical scales and the NLR. Thus, we adopted non-parametric tests. Categorical data were compared with the chi-square test or Fisher’s test. Between groups comparisons were performed with the Mann–Whitney U test, while for intra-group comparison we adopted the Wilcoxon test. Correlations were assessed with the Spearman test.

To evaluate the association between presence of SII and the changes in clinical scales scores between T_0_ and T_1,_ we used a non-parametric model for repeated measures with two factors^38^: factor TIME (within subjects, 2 levels: T_0_ vs. T_1_, expression of modification in clinical scales score in the overall study population) and factor GROUP (between subjects, 2 levels: SII vs. IC, expression of the overall between-groups difference). The TIMExGROUP interaction was assessed to test whether the extent of improvement between T_0_ and T_1_ differed between SII and IC groups. The non-parametric model was always corrected for age, sex, time from stroke onset to NRB admission, and stroke volume. Only in the presence of a significant interaction, a post-hoc intra-group analysis with the Wilcoxon test was performed. A Bonferroni correction was applied to all tests to correct for multiple comparisons.

We performed a linear regression to test which variables were associated with the FIM score at hospital discharge (primary outcome).

The level of significance was set at α = 0.050 for all statistical analyses.

### Supplementary Information

Supplementary Information.

## Data Availability

The dataset generated and/or analysed during the current study is available in the Zenodo repository (10.5281/zenodo.10057752). The dataset is available from the corresponding author on reasonable request.

## Abbreviations

SII: Stroke-induced immunosuppression defined as NLR ≥ 5 at admission
IC: Immunocompetence defined as NLR < 5
ICH: Primary spontaneous intracerebral haemorrhage
IS: Ischemic stroke
NLR: Neutrophil-to-lymphocyte ratio
NRB: Neurorehabilitation
T_0_: Neurorehabilitation unit admission
T_1_: Neurorehabilitation unit discharge
