# Supplementary material for: The role of stroke-induced immunosuppression as a predictor of functional outcome in the neurorehabilitation setting
Source: Sci Rep. 2024 Apr 9;14:8320. doi: 10.1038/s41598-024-58562-1 (PMC11003970; doi:10.1038/s41598-024-58562-1)
Supplement: Supplementary file 1 — Supplementary Information. [file 41598_2024_58562_MOESM1_ESM.docx]

**Supplemental Material**

The role of Stroke-Induced Immunosuppression as a predictor of functional outcome in the neurorehabilitation setting

**Authors:**

Gloria Vaghi^1,2^, MD; Andrea Morotti^3,4^, MD, PhD; Elisa Maria Piella^1,2^, MD; Micol Avenali^1,2^, MD, PhD; Daniele Martinelli^2^, MD, PhD; Silvano Cristina^2^, MD; Marta Allena^2^, MD, PhD; Valentina Grillo^1,2^, BS; Michele Corrado^1,2^, MD; Federico Bighiani^1,2^, MD; Francescantonio Cammarota^1,2^, MD; Alessandro Antoniazzi^1,2^, MD; Federica Ferrari^1,5^, MD, PhD; Federico Mazzacane^1,5^, MD; Anna Cavallini^1,5^, MD; Anna Pichiecchio^1,6^, MD; Elisa Rognone^6^, MD; Luca Martinis^7,2^, BS; Luca Correale^7^, BS, PhD; Stefano Filippo Castiglia^8,9^, BS; Dante Trabassi^8^, BS; Mariano Serrao^8,9^, MD, PhD; Cristina Tassorelli^1,2^, MD, PhD; Roberto De Icco^1,2^, MD PhD

**Supplementary material 1**

**Neurorehabilitation programme**

According to Italian and regional law (n° X/1980, 20th June 2014), all patients received daily sessions with a physiotherapist lasting approximately 90 minutes, up to 500 minutes/week across 6 days per week. The overall neurorehabilitation programme lasted between two to eight weeks and was tailored according to patients needs and functional status. Adjunctive treatments with speech therapists, psychologists or occupational therapists were performed up to 150 minutes/week across 5 day per week.

All enrolled patients underwent a standardized in-hospital rehabilitation programme mainly based on the Bobath approach ^1^.

Within the first 24 hours from admission all patients underwent a thorough functional assessment, including administration of outcome scales.

In the early phase of the in-hospital rehabilitative process, the physiotherapy program mainly focused on mobilization of specific body segments, somatosensory afferents enhancement, motor and cognitive stimulation, management of neurovegetative problems, progressive re-education to the sitting position, and re-education to postural transitions and transfers. For bedridden patients, frequent postural changes were performed approximately every 2 hours.

As soon as possible, patients started a more structured, active and integrated programme in a dedicated and equipped space. The daily rehabilitative programme included: passive and active mobilization, manual techniques for management of spasticity, integration of the affected hemisome into postural activities, integration of the affected upper limb into task-specific activities, early verticalization, balance training, walking and gait rehabilitation of progressive intensity, and cardio-vascular reconditioning. Use of rehabilitative aids (namely canes, crutches, rollators, walkers, wheelchairs) was adopted according to patients’ needs.

1. Pathak, A., Gyanpuri, V., Dev, P. & Dhiman, N. The Bobath Concept (NDT) as rehabilitation in stroke patients: A systematic review. *J Fam Med Prim Care*. 10(11), 3983, DOI:10.4103/jfmpc.jfmpc_528_21 (2021).

**Supplementary Table**

**Supplementary Table 1:** Infectious complication during hospitalization in the Neurorehabilitative Unit and destination at hospital discharge.

|  | | Total | IC group | SII group | p-value |
| --- | --- | --- | --- | --- | --- |
| Number of patients | | 96 | 81 | 15 | - |
| INFECTIOUS COMPLICATION | | | | | |
| At least one infectious complication | | 33 (34.4%) | 21 (25.9%) | 12 (80%) | **0.001** |
| Pneumonia | | 6 (6.3%) | 4 (4.9%) | 2 (13.3%) | 0.235 |
| Urinary tract infection | | 23 (24%) | 15 (18.5%) | 8 (53.3%) | **0.007** |
| Sepsis | | 4 (4.2%) | 2 (2.5%) | 2 (13.3%) | 0.114 |
| Other | | 5 (5.2%) | 4 (4.9%) | 1 (6.7%) | 0.581 |
| DYSPHAGIA and URINARY DISORDERS at NRB DISCHARGE (T_1_) | | | | | |
| Dysphagia | | 28 (29.2%) | 20 (24.7%) | 8 (53.3%) | **0.004** |
| Urinary disorders | | 41 (42.7) | 34 (41.9%) | 7 (46.7%) | 0.256 |
| DESTINATION AT HOSPITAL DISCHARGE | | | | | |
| Days of hospitalization | | 47.2 ± 14.9 | 46.4 ± 15.1 | 51.1 ± 13.8 | 0.081 |
| Destination at hospital discharge | Home | 71 (74.0%) | 64 (79.0%) | 7 (46.7%) | **0.032** |
|  | Non-intensive rehabilitation | 13 (13.5%) | 9 (11.1%) | 4 (26.7%) |  |
|  | Acute department | 12 (12.5%) | 8 (9.9%) | 4 (26.7%) |  |

*Legend:* SII: stroke-induced immunosuppression. NRB: Neurorehabilitation Unit. IC: Immunocompetent patients. SII is defined as a neutrophil to lymphocyte ratio (NLR) ≥ 5.
